# Reconstitution of *C9orf72* GGGGCC repeat-associated non-AUG translation with purified human translation factors

**DOI:** 10.1038/s41598-023-50188-z

**Published:** 2023-12-20

**Authors:** Hayato Ito, Kodai Machida, Mayuka Hasumi, Morio Ueyama, Yoshitaka Nagai, Hiroaki Imataka, Hideki Taguchi

**Affiliations:** 1 School of Life Science and Technology, Tokyo Institute of Technology, S2-19, Nagatsuta 4259, Midori-ku, Yokohama, 226-8501 Japan; 2 Cell Biology Center, Institute of Innovative Research, Tokyo Institute of Technology, S2-19, Nagatsuta 4259, Midori-ku, Yokohama, 226-8501 Japan; 3 Graduate School of Engineering, University of Hyogo, Shosha, 2167, Himeji, Hyogo 671-2280 Japan; 4 Department of Neurology, Faculty of Medicine, Kindai University, Ohonohigashi 377-2, Osaka-Sayama, 589-8511 Japan

**Keywords:** Translation, Ribosome, Amyotrophic lateral sclerosis

## Abstract

Nucleotide repeat expansion of GGGGCC (G_4_C_2_) in the non-coding region of *C9orf72* is the most common genetic cause underlying amyotrophic lateral sclerosis and frontotemporal dementia. Transcripts harboring this repeat expansion undergo the translation of dipeptide repeats via a non-canonical process known as repeat-associated non-AUG (RAN) translation. In order to ascertain the essential components required for RAN translation, we successfully recapitulated G_4_C_2_-RAN translation using an in vitro reconstituted translation system comprising human factors, namely the human PURE system. Our findings conclusively demonstrate that the presence of fundamental translation factors is sufficient to mediate the elongation from the G_4_C_2_ repeat. Furthermore, the initiation mechanism proceeded in a 5′ cap-dependent manner, independent of eIF2A or eIF2D. In contrast to cell lysate-mediated RAN translation, where longer G_4_C_2_ repeats enhanced translation, we discovered that the expansion of the G_4_C_2_ repeats inhibited translation elongation using the human PURE system. These results suggest that the repeat RNA itself functions as a repressor of RAN translation. Taken together, our utilization of a reconstituted RAN translation system employing minimal factors represents a distinctive and potent approach for elucidating the intricacies underlying RAN translation mechanism.

## Introduction

The abnormal expansion of specific nucleotide repeats (repeat expansion) within the genome has been established as a causative factor in neurodegenerative diseases such as amyotrophic lateral sclerosis (ALS) and spinocerebellar ataxia (SCA)^1,2^. Recent investigations have revealed a noncanonical translation mechanism for transcripts containing repeat expansions, termed repeat-associated non-AUG (RAN) translation, which operates independently of the conventional initiation codon AUG^3–9^. Notably, a representative instance of RAN translation occurs at the GGGGCC (G_4_C_2_) repeat located in the first intron of *C9orf72* (Fig. S1) (referred to as C9-RAN), constituting the most prevalent genetic mutation observed in familial ALS^10,11^. C9-RAN involves the translation of all conceivable frames of the G_4_C_2_ repeat, which is transcribed in both directions. Specifically, the sense strand (GGGGCC) yields dipeptide repeats (DPRs) consisting of Gly-Ala (GA from GGG-GCC, 0 frame), Gly-Pro (GP from GGG-CCG, + 1 frame), and Gly-Arg (GR from GGC-CGG, + 2 frame). Indeed, these DPRs have been detected not only in patient tissues^4,6,12,13^, but also in diverse model organisms, and their association with cytotoxicity is well-documented^4,6,13–20^. Based on these recent findings, it is proposed that C9-RAN may assume a central role in the pathogenesis of repeat expansion-associated ALS, offering a novel avenue to explore potential therapeutic targets for the disease.

The translation process consists of four fundamental steps: initiation, elongation, termination, and ribosome recycling^21^. In eukaryotes, the canonical initiation of translation takes place through a scanning mechanism mediated by the cap structure at the 5′-end of the mRNA^21–23^. This scanning mechanism is strictly regulated by over ten translation initiation factors in conjunction with the eIF4F complex, comprising eIF4A, eIF4G, and eIF4E, which initiates translation from the canonical AUG initiation codon. Recent genome-wide studies have unveiled diverse instances of non-AUG translation, including those initiated from CUG and GUG codons^24–26^. Non-AUG translation is believed to involve eIF2A and eIF2D, alternative factors for eIF2^27–30^. Furthermore, it is recognized that certain eukaryotic mRNA and some genes within viral genomes commence translation without scanning, utilizing a sophisticated RNA structure element termed an internal ribosomal entry site (IRES)^23,31^.

The initiation mechanism of C9-RAN exhibits commonalities with canonical mechanisms. For example, previous studies utilizing monocistronic reporters have shown that C9-RAN initiates from near cognate codons, such as CUG and AGG, located upstream of the G_4_C_2_ repeat sequence, through a scanning mechanism involving eIF4A^32–34^. However, contrasting findings employing bicistronic reporters have indicated that C9-RAN commences independent of the cap structure^35,36^. Furthermore, diverse mechanisms generating multiple translational frames have been reported, such as ribosomal frameshift during elongation on the repeat sequence and initiation from distinct start codons for each frame^33,34,37–42^. The occurrence of frameshift in C9-RAN is predicted to be associated with a secondary structure of the mRNA, namely the guanine quadruplex (G4-RNA), formed as a consequence of the guanine-rich property of the G_4_C_2_ repeat^33^. In addition, it has been observed that C9-RAN is regulated by factors that are not essential for canonical translation. Notably, eIF2A, an alternative factor for eIF2, has been postulated to modulate C9-RAN, although its requirement in transfected cells remains a topic of controversy^35,43,44^. Indeed, recent studies have provided evidence that ribosomes can be stalled by positively charged peptides translated by C9-RAN and by the repeat RNA itself^45–48^. These stalling events are proposed to play a critical role in inducing ribosome-associated quality control (RQC) pathway.

Hence, numerous discussions ensue regarding the molecular mechanism of C9-RAN, primarily attributed to the lack of consistent findings concerning the fundamental process of translation and factors associated with C9-RAN. The utilization of cell-based experimental systems, such as transfected cells and cell lysate translation systems, is believed to contribute to these inconsistencies. Such cell-based systems encompass proteins unrelated to translation and exhibit varying quantities of translation factors contingent upon cell types^49^, thereby yielding a heterogeneous array of results, occasionally conflicting in nature. Indeed, GP frames occurring in C9-RAN have been shown to exhibit different translation efficiencies depending on the cell type^32,33^. Consequently, to ascertain the intricate molecular mechanism of C9-RAN comprehensively, the employment of an in vitro translation system independent of cell lysate becomes imperative.

**Figure 1:**
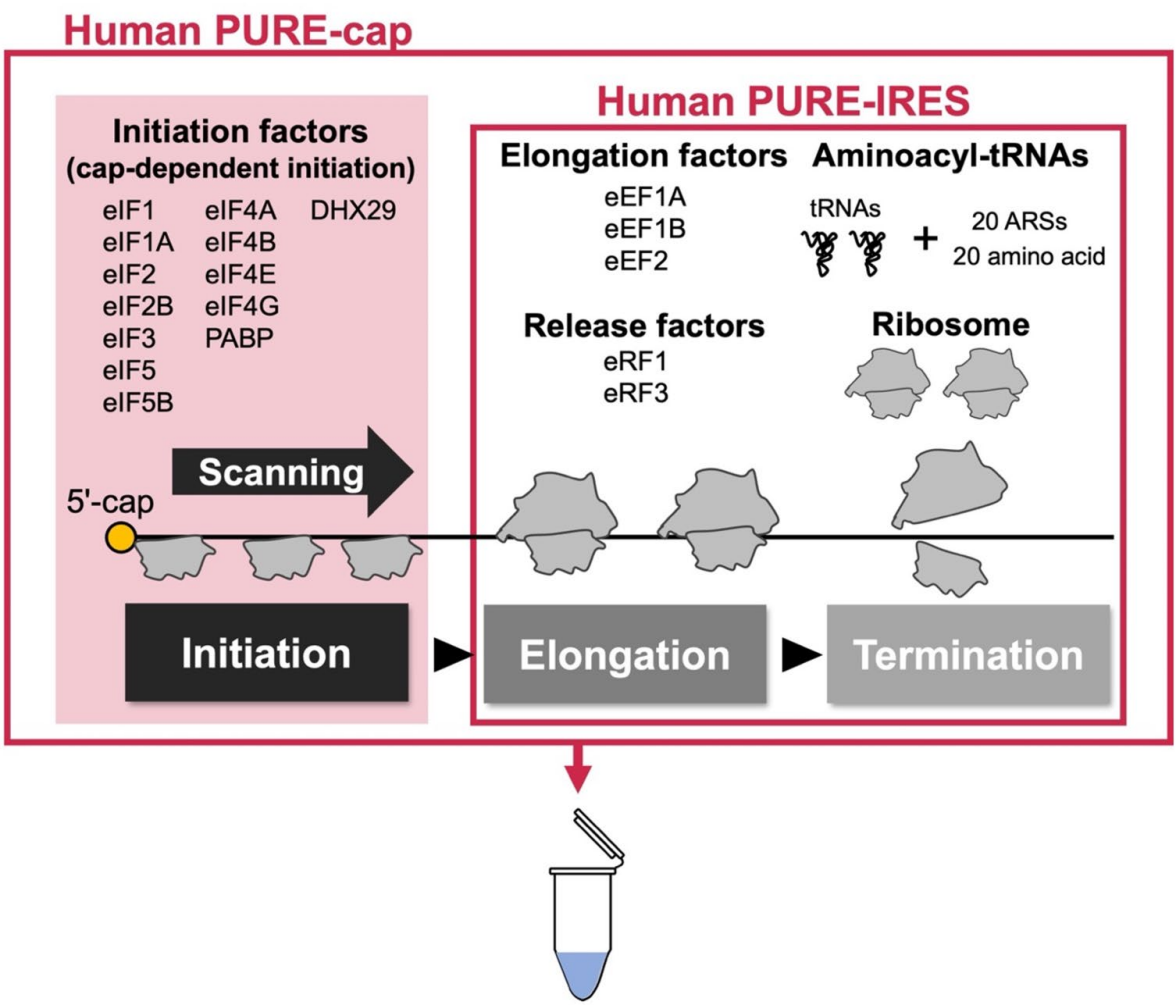
A schematic depiction of the human PURE system. Human PURE-IRES consists of two elementary steps in translation: namely elongation and termination. Human PURE-cap includes eukaryotic initiation factors (eIFs) responsible for scanning-mediated translation initiation, in addition to human PURE-IRES.

To circumvent such complexities associated with C9-RAN and elucidate its fundamental, the adoption of a reductionist approach is invaluable. In this context, a cell-free translation system reconstituted in vitro, employing purified factors indispensable for translation, serves as the optimal experimental system for conducting a comprehensive investigation of the mechanism governing C9-RAN. Subsequent to the establishment of a reconstituted translation system in *Escherichia coli*^50^, recent advancements have led to the development of eukaryotic translation factor-based reconstituted translation systems^51–53^. Within the human factor-based reconstituted translation system (human PURE, Fig. 1), a reconstituted system using IRES (human PURE-IRES)^51^, thereby omitting initiation factors, was first developed. This was succeeded by the development of a fully reconstituted system, enabling translation initiation in a cap-dependent manner in the presence of initiation factors (human PURE-cap)^52^.

In this study, we successfully recapitulated C9-RAN using the human PURE system. By employing distinct modes of translation, namely IRES-dependent and cap-dependent, we were able to distinguish and investigate the elementary processes underlying C9-RAN. Through the utilization of this minimalist system, we acquired valuable insights into translation elongation and initiation within the context of C9-RAN. Furthermore, we observed the suppression of C9-RAN with longer repeat RNA in human PURE. These findings shed light on previously ambiguous aspects of the molecular mechanism underlying C9-RAN, highlighting the merits of employing the bottom-up approach facilitated by human PURE as a versatile tool for dissecting RAN translation associated with other diseases.

## Results

### G_4_C_2_ translation elongation with minimal translation factors

**Figure 2:**
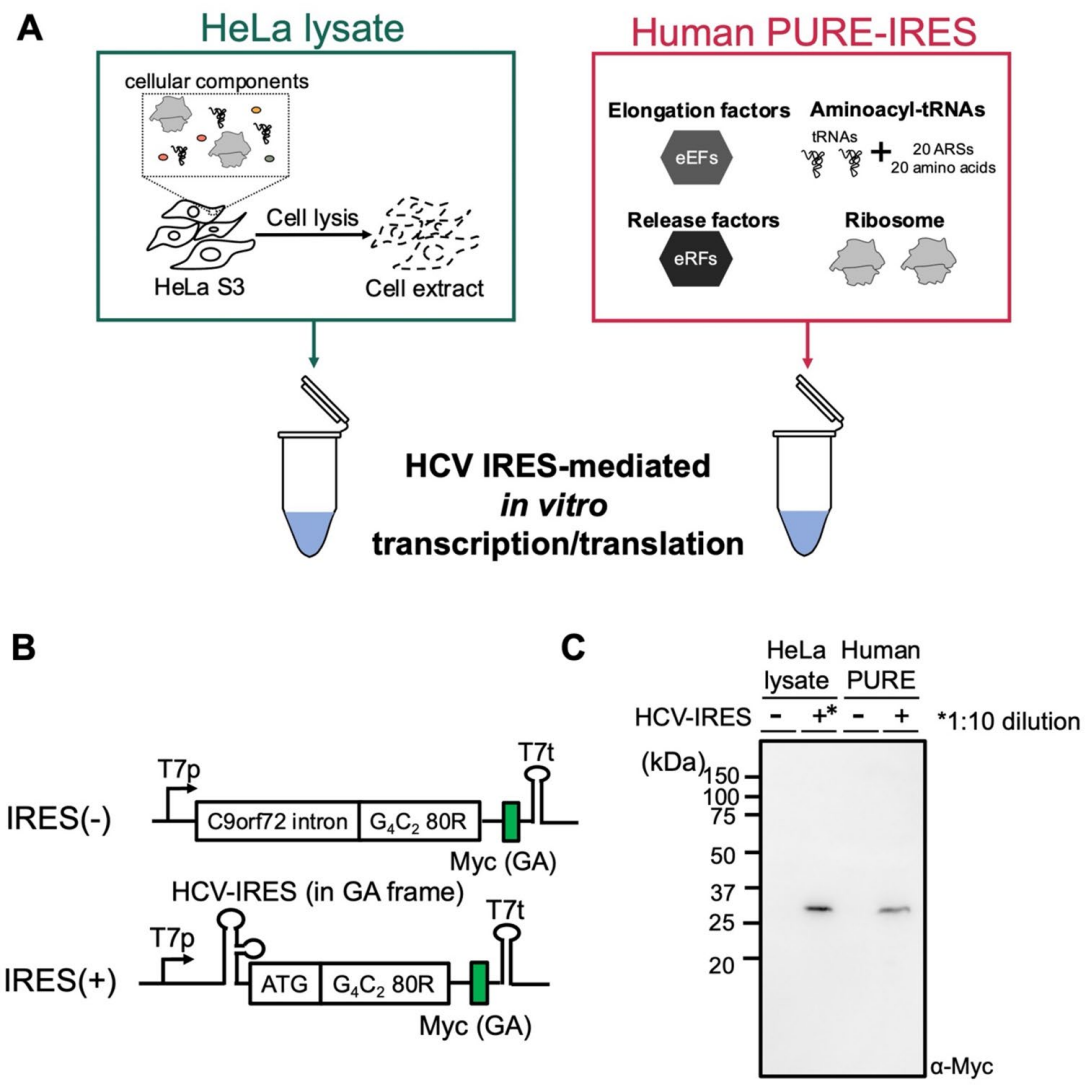
Minimal translation factors are sufficient for the translation of the G_4_C_2_ repeats. (**A**) Illustration of each in vitro translation system. HeLa lysate^71^ contained intracellular factors, while the human PURE-IRES system utilized purified translation factors^51^. Hepatitis C Virus (HCV)-IRES, enabling translation initiation without initiation factors^54^, was employed to investigate the elongation mechanism of the G_4_C_2_ repeats. (**B**) Schematic representation of the construct for monitoring the elongation of the G_4_C_2_ repeats. HCV-IRES was inserted upstream of the G_4_C_2_ repeat sequence, with a Myc-tag introduced downstream of the G_4_C_2_ repeat in the GA frame. (**C**) Anti-Myc western blot of the G_4_C_2_ repeat reporter plasmids expressed in each in vitro translation system. To prevent band detection saturation in HeLa lysate, IRES(+) reaction was diluted 1:10 in the sample buffer, as indicated by the asterisk (*).

To investigate the elementary steps involved in C9-RAN, we employed human PURE system, including purified ribosomes, eukaryotic initiation/elongation/release factors (eIFs, eEFs, and eRFs)^51,52^ (Figs. 1, 2A). Initially, we aimed to determine whether the G_4_C_2_ repeat sequences could undergo elongation with the minimal translation elongation factors. For this purpose, we used the hepatitis C virus (HCV) IRES to recruit the ribosome in the absence of eIFs^54^. We introduced the HCV IRES, along with the ATG codon following the IRES, upstream of the GA frame (GGG-GCC/0 frame) within the G_4_C_2_ 80 repeat (80R) sequence without *C9orf72* intron (Fig. 2B) as previous studies have indicated that the translation product derived from the GA frame is the most abundant in C9-RAN^6,32,33^. We conducted translation experiments using a fusion gene encoding HCV-IRES-ATG-G_4_C_2_-80R, followed by a Myc-tag for detection, in HeLa lysate and the human PURE-IRES. Moreover, to demonstrate the HCV-IRES-dependent initiation of translation, a translation reaction was conducted employing a *C9orf72* intron sequence while omitting the HCV-IRES element. This reporter sequence lacks the capability to provide a 5′ cap and thus does not induce intron-derived cap-dependent initiation of RAN translation. We detected translation products of the expected molecular weight (~ 25 kD for ATG-G_4_C_2_ 80R-Myc) in both HeLa lysate and the human PURE-IRES, only when IRES was present (Fig. 2C), although the translation product yield was considerably smaller in the human PURE-IRES. Given that this experiment forced the translation initiation to the IRES-linked ATG codon, this translation does not meet the definition of RAN translation. Nevertheless, this experiment is important in that it extracted only the elongation step in translation and demonstrates that the G_4_C_2_ repeat can undergo elongation with minimal elongation factors.

### Ribosomal frameshifting during elongation in the GA (0) frame

**Figure 3:**
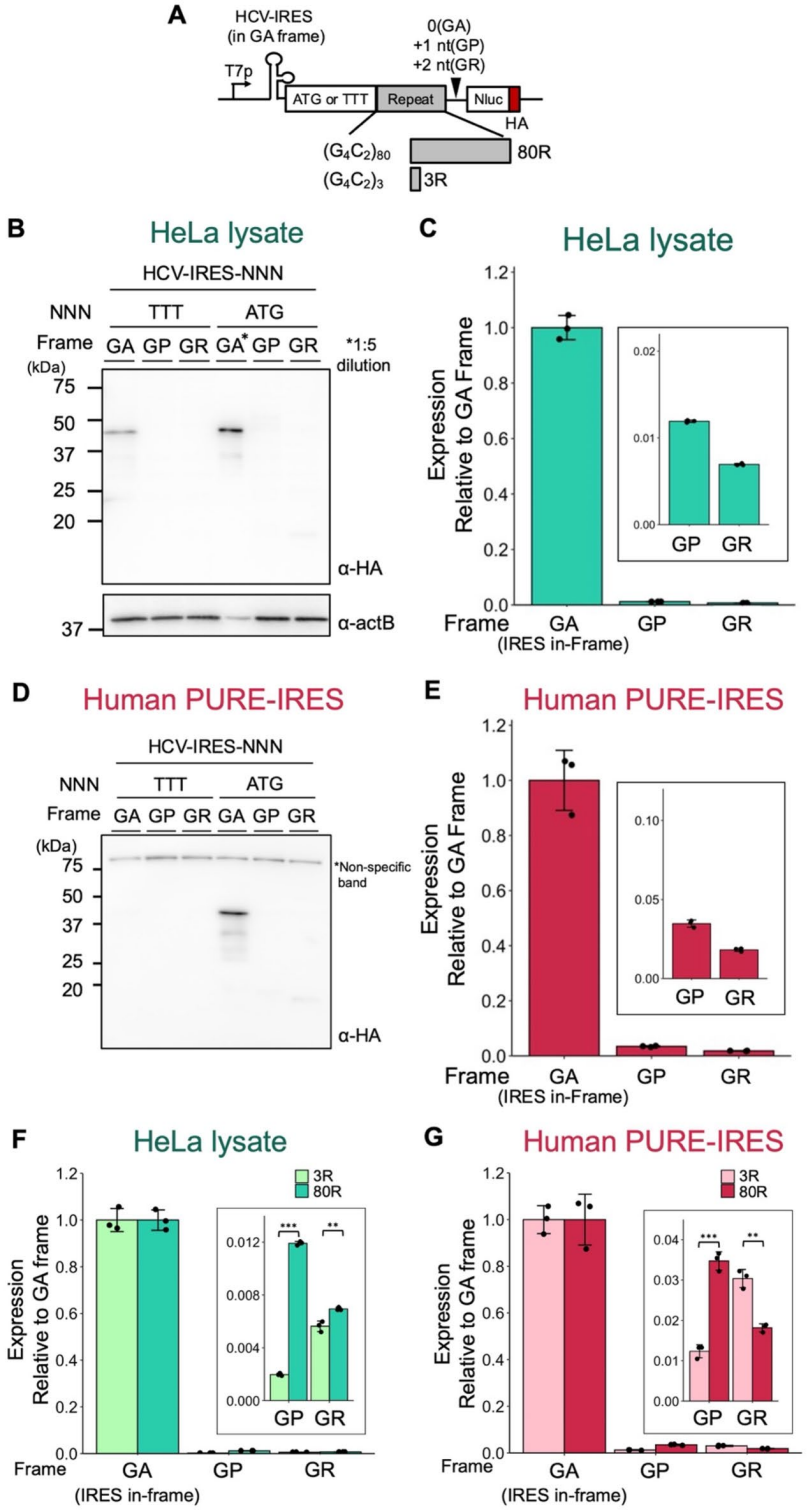
Ribosomal frameshift in the GA (0) frame. (**A**) Schematic representation of the elongation reporters for the G_4_C_2_ repeats. Nano-Luciferase (Nluc) was used as a reporter enzyme to quantify translation efficiency. HA-tag in the GA-frame was introduced downstream of the G_4_C_2_ repeat. (**B**, **D**) Anti-HA western blot of the elongation-reporter plasmids expressed in each in vitro translation system. (**B**) HeLa lysate, (**D**) human PURE-IRES. (**C**, **E**) Relative expression from IRES-ATG-(G_4_C_2_)_80_-Nluc-HA normalized to the GA frame. (**C**) HeLa lysate, (**E**) human PURE-IRES. (**F**–**G**) Expression of IRES-ATG-(G_4_C_2_)_3_-Nluc and IRES-ATG-(G_4_C_2_)_80_-Nluc normalized to their respective GA frames. (**F**) HeLa lysate, (**G**) human PURE-IRES. Error bars represent standard deviations (± SD) from three technical replicates. Two-tailed student’s *t* test, **p ≦ 0.01, ***p ≦ 0.001. Raw data of the Nluc assay are provided in Table S1.

Next, we established a nano-luciferase (Nluc) reporter assay system to quantitatively measure the translation products with high sensitivity. Nluc and an HA-tag were introduced downstream of the IRES-ATG-(G_4_C_2_)_80_ or IRES-TTT-(G_4_C_2_)_80_ genes (Fig. 3A). TTT codon was used as a control for ATG start codon. In addition to the GA (0) frame, we generated constructs with two additional reading frames: the GP (+ 1) frame and the GR (+ 2) frame, achieved by inserting one or two nucleotides, respectively, following the (G_4_C_2_)_80_ repeat. Western blotting analysis following the translation of the Nluc constructs using the human PURE-IRES or HeLa lysate demonstrated an ATG-dependent expression of the GA frame product compared to the products from other frames (Fig. 3B, D). The ATG-dependency, along with the approximate expected molecular weight for the ATG-(G_4_C_2_)_80_-Nluc-HA gene (~ 39 kD), indicates that translation elongation commences from the ATG immediately after the IRES. Note that translation of the TTT-containing gene using the HeLa lysate yielded a small amount of the 39 kD protein in the GA frame (Fig. 3B).

We first confirmed that luciferase activity-based translation was entirely dependent on the IRES in both HeLa lysate and the human PURE-IRES translation (Fig. S2A). Subsequently, we reproducibly observed luciferase activities across all frames, including the minor GP (+ 1) and GR (+ 2) frames, during the translation of the genes containing either ATG or TTT start codons, using both HeLa lysate and the human PURE-IRES (Figs. 3C, E, S2B, C). Earlier studies employing lysate-based translation also showed translation occurring in all frames^32,33^, with the GP and GR frames suggested to result from ribosomal frameshifting originating from the GA frame^33,39^. Prior studies have shown that the repeat RNA itself, responsible for generating C9-RAN, functions as an IRES to drive C9-RAN translation^35,36,38,55,56^. If the repeat RNA primarily acts as an IRES, the contribution of ATG in translation in the IRES-ATG construct would be small, i.e., one would expect that IRES-ATG and IRES-TTT would have similar amounts of translation. However, this is not the case; we observed a substantial reduction in translation (~ 1/100) upon changing ATG to TTT (Fig. S2B, C, Table S1). This striking difference strongly suggests that the primary translation initiation is from IRES-ATG, with little, if any, contribution from within the repeat RNA. Through a series of experiments using the human PURE-IRES, our observation of translation initiation from the ATG or TTT start codons immediately after the IRES (Figs. 3D, E, S2C), along with the minimal translation activity in the absence of IRES (Fig. S2A), led us to suggest the occurrence of frameshifting even within a simplified translation system. The luciferase activities of the GP and GR frames relative to the GA frame in ATG-mediated human PURE-IRES translation were 3.5 and 1.9%, respectively (Fig. 3E), higher than those observed in the HeLa lysate translation (1.2 and 0.7% for GP and GR, respectively, Fig. 3C). This suggests the possible presence of a frameshift repressor in the lysate.

Then, we examined the potential influence of the G_4_C_2_ repeat length on the translation of GP (+ 1) and GR (+ 2) frames in the human PURE-IRES, considering that a prior study using lysate translation demonstrated that frameshifting on the G_4_C_2_ repeat sequence is enhanced by longer repeat RNA^33^. Within the human PURE-IRES, translation of the GP frame exhibited an increase, while the GR frame displayed a decrease in response to longer repeat lengths (Fig. 3G), thus indicating that the length of the repeat exerts an influence on frameshifting.

### Translation initiation in C9-RAN can be initiated using minimal initiation factors

**Figure 4:**
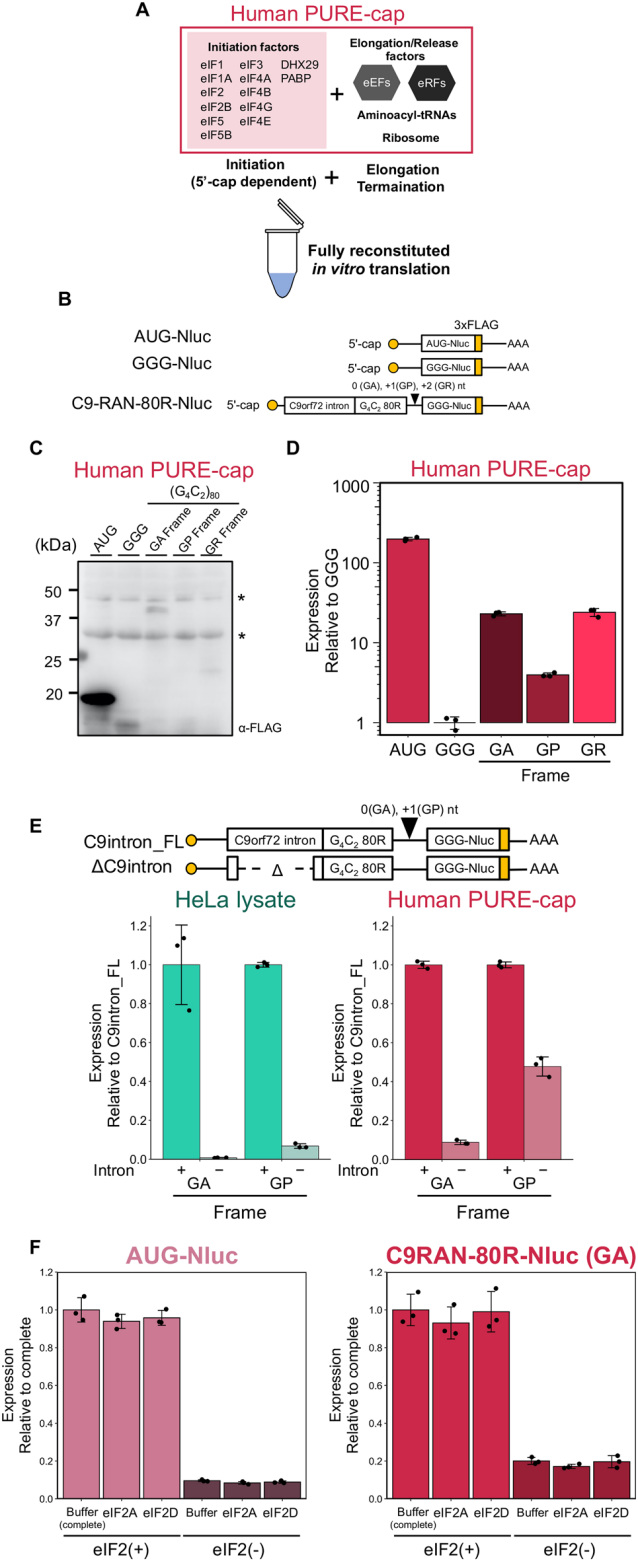
Minimal translation factors are enough for initiating C9-RAN translation. (**A**) Illustration depicting the human PURE-cap system^52^. (**B**) Diagram presenting the C9-RAN reporters based on previously reported designs^32^. Nluc was used as a reporter enzyme to quantify translation efficiency. A FLAG-tag was inserted downstream of the G_4_C_2_ repeat in each frame. (**C**) Anti-FLAG western blot of the C9-RAN reporter plasmids expressed in human PURE-cap. An asterisk (*) indicates a non-specific band. (**D**) Relative expression of the C9-RAN reporters normalized to GGG-Nluc in human PURE-cap. Error bars represent ± SD from three technical replicates. Raw data of the Nluc assay are provided in Table S2. (**E**) Relative expression of the C9-RAN reporters with a variant intron in each in vitro translation system. Normalized *C9orf72* intron full length (FL). (**F**) Relative expression of the AUG-Nluc and C9-RAN reporters in the presense (eIF2(+)) and the absence (eIF2(−)) of eIF2 in Human PURE. Under each condition, eIF2A and eIF2D were added. The ratio was normalized with complete human PURE (eIF2(+)).

Subsequently, we investigated the initiation mechanism of C9-RAN by employing a fully reconstituted 5′ cap-dependent translation system equipped with canonical thirteen eIFs (referred to as human PURE-cap, Fig. 4A)^52^. In order to quantify C9-RAN via a scanning mechanism, we constructed a series of reporter mRNAs, utilizing the methodology previously developed by Green et al.^32^. Initially, we evaluated the property of human PURE-cap in non-AUG translation, employing mRNAs containing CUG or AGG codons fused with Nluc. We observed a relatively higher translation efficiency for the CUG codon compared to that observed in HeLa lysate (Fig. S3). Then, we proceeded to translate a reporter mRNA harboring a 5′-cap, the *C9orf72* intron, (G_4_C_2_)_80_ repeats, followed by Nluc and a FLAG-tag (Fig. 4B). Through western blotting analysis, we confirmed the synthesis of ~ 40 kD protein bands corresponding to the GA frame in both the human PURE-cap system and HeLa lysate (Figs. 4C, S4A). The approximate molecular weight of these protein bands aligns with the expected size (35 kD) if translation was initiated from the upstream sequence of the repeats (Fig. S1), as previously demonstrated^32–35^. This result, obtained using the human PURE-cap system, provides evidence that C9-RAN can be initiated utilizing minimal translation factors. Additionally, translation in HeLa lysate produced a ~ 23 kD protein band in the GR frame (Fig. S4A). The molecular weight of ~ 23 kD corresponds well with the calculated size (23.5 kD) of the translation product when initiation occurs at the downstream AUG codon within the G_4_C_2_ repeats in the GR frame, indicating that this product likely represents a leaky scanning product (Fig. S4B).

Luciferase activities were detected in all frames during both HeLa lysate and human PURE-cap translation, strictly dependent on the presence of a cap structure (Figs. 4D, S4C, S5A), thereby confirming the sufficiency of minimal translation factors for C9-RAN. However, the overall translation level of C9-RAN in the human PURE was lower compared to that in HeLa lysate. For instance, the translation of the GA frame in HeLa lysate was comparable to that of AUG-Nluc mRNA (Fig. S4C), whereas it constituted approximately one-tenth of the translation observed in human PURE-cap (Fig. 4D). This discrepancy suggests that cap-dependent translation using the PURE system requires additional factors to enhance the efficiency of translation.

Moreover, prior investigations have revealed that the start codon of the GA frame is CUG^32–35^. Upon introducing mutations to this CUG, such as AUG and GGG, translation was significantly reduced in the case of GGG, observed in both HeLa lysate and human PURE (Fig. S5B). However, a notable level of translation persisted at the GGG codon. To explore the potential existence of other translation initiation sites, we proceeded to delete the intronic region upstream of the repeats, thereby introducing a stop codon in each frame (Fig. 4E). Subsequent analysis showed that the GA frame exhibited minimal translation in HeLa lysate, while a certain level of translation persisted in human PURE. Intriguingly, the GP frame displayed detectable translation in both HeLa lysate and human PURE, even after deletion of the intronic region. This finding supports the notion that the GA and GP frame can be translated from within the repeats in a scanning-dependent manner in human PURE.

Previous studies have shown that C9-RAN involves nonessential translation initiation factors such as eIF2A and eIF2D^35,43,44^. However, some reports have also shown that they are dispensable, making it ambiguous whether eIF2A or eIF2D directly affect C9-RAN^43^. To explore the direct involvement of eIF2A and eIF2D in initiation of C9-RAN, we prepared a customized human PURE system. These were configured by eliminating eIF2 from the complete human PURE system and subsequently incorporating the respective factors. Furthermore, we prepared PURE systems containing these factors alongside eIF2, envisioning their potential synergy in the elongation mechanism. For each, translation reactions were performed with canonical AUG codons and C9-RAN reporters to verify C9-RAN specificity. In the absence of eIF2, the efficacy of C9-RAN translation was notably reduced similar to conventional AUG translation, even when eIF2A or eIF2D was introduced (Fig. 4F). Moreover, in the presence of eIF2, the addition of eIF2A or eIF2D did not yield a discernible enhancement in C9-RAN efficiency (Fig. 4F). This observation signifies that eIF2A and eIF2D are not directly implicated in C9-RAN. These findings collectively imply that C9-RAN fundamental proceeds with minimal essential translation factors, including eIF2, and does not necessitate the direct participation of eIF2A or eIF2D.

### C9-RAN translation is suppressed by longer G_4_C_2_ repeats in human PURE translation

**Figure 5:**
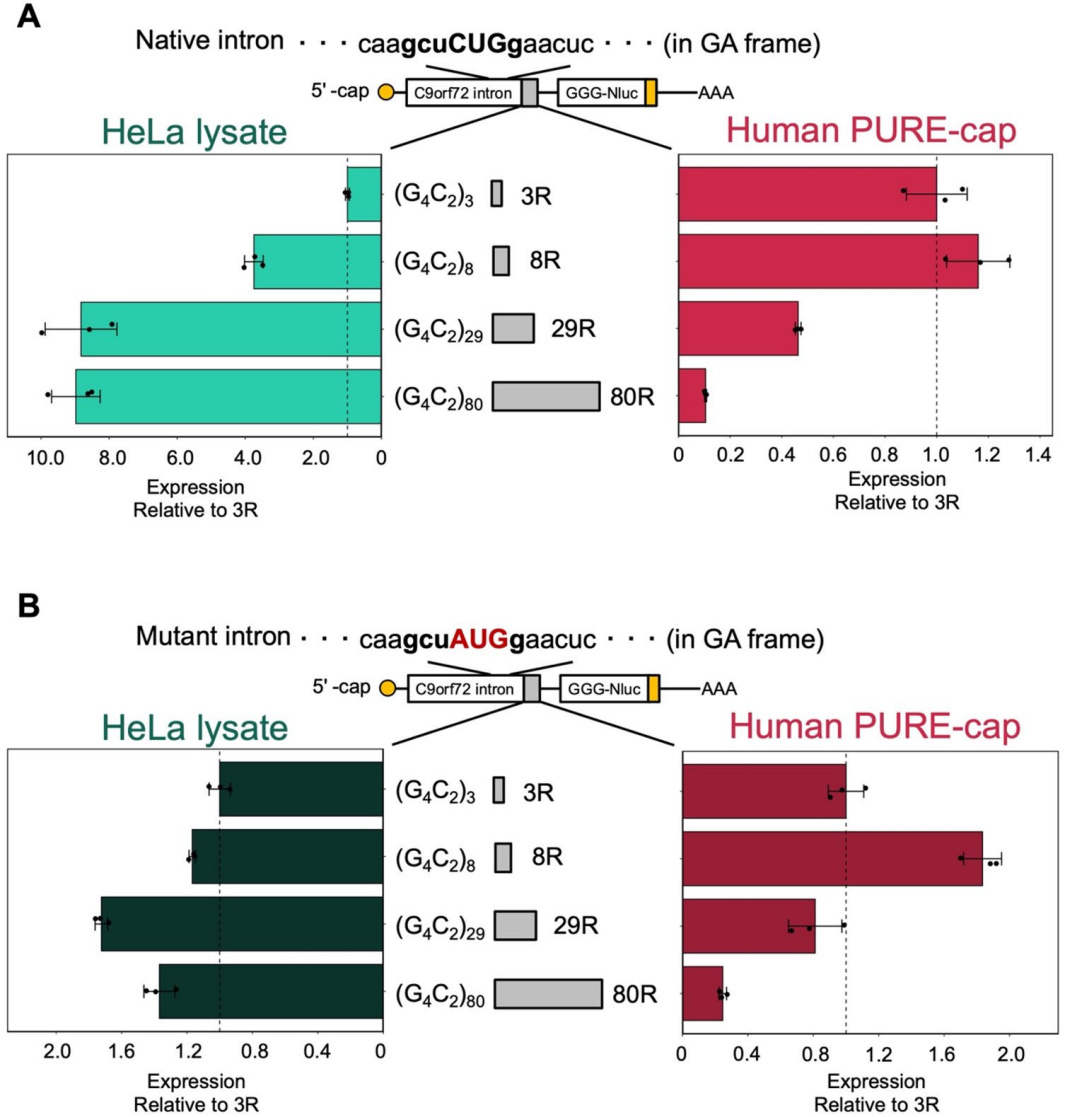
Inhibition of C9-RAN by longer repeat RNA in human PURE-cap. (**A**, **B**) Relative expression levels of C9-RAN reporters with varying numbers of repeats in each in vitro translation system. Normalized to 3R-Nluc. (**A**) The first intron of *C9orf72*. (**B**) Mutant intron substituting CUG with AUG codon. The CUG codon located -24 nt upstream of the repeat sequence is predicted as the GA frame start codon^32–35^. Error bars represent ± SD from three technical replicates.

Enhancement of RAN translation can be attributed to longer repeat RNA sequences^3,32–35,57,58^. Given the lack of comprehensive understanding regarding the underlying mechanism, we investigated the dependence of repeat-length in human PURE translation (Fig. 5A). In the HeLa lysate translation, the augmentation of C9-RAN occurred in accordance with the length of G_4_C_2_ repeats in both GA and GP frames (Figs. 5A, S6A). This enhancement reached saturation at 29 repeats, exhibiting an approximately nine-fold increase in comparison to the three-repeat scenario in the GA frame (Fig. 5A). Conversely, in contrast to the HeLa lysate translation, the utilization of human PURE-cap translation inhibited C9-RAN from 29 repeats, leading to decline of less than 10% in the GA frame translation when utilizing 80 repeats (Fig. 5A). The inhibition of repeat length was also observed in the GP frame during human PURE-cap translation (Fig. S6A).

We subsequently investigated whether the repeat RNA length-dependent translational repression observed in the human PURE system resulted from issues related to non-AUG initiation or elongation of repeat RNA^27–30^. To investigate this, we introduced a CUG-to-AUG substitution in the *C9orf72* intron. Interestingly, this substitution nearly abolished the length-dependent enhancement observed in the HeLa lysate translation (Fig. 5B). However, in human PURE-cap, the CUG-to-AUG substitution did not alter the declining trend in translation efficiency observed beyond 29 repeats (Fig. 5B). It is also plausible that C9-RAN undergoes suppression as a result of the depletion of specific tRNAs, possibly due to the continuous translation of specific amino acid sequences within the human PURE. To investigate the possibility, we conducted a parallel experiment involving the GGG-CCU repeat in NOP56-RAN translation of SCA36^39^. One of the frames in the NOP56-RAN translation produces GP frame (GGG-CCU), which is the same as one of the frames in C9-RAN. Notably, we observed no translational repression caused by extended repeats in the case of NOP56 (Fig. S6B). Taken together, these results suggest that the longer repeat RNA-mediated suppression of C9-RAN in human PURE-cap is not solely attributed to an anomaly in non-AUG initiation and consecutive amino acid sequence.

## Discussion

In this study, we successfully recapitulate RAN translation from *C9orf72*-(G_4_C_2_) repeat (C9-RAN) using a reconstituted translation system comprised of purified human translation-related factors (human PURE). The pivotal and straightforward message conveyed by this study is the explicit demonstration that C9-RAN occurs exclusively through the utilization of canonical translation factors. We particularly emphasize the unique advantage of human PURE in dissecting elementary steps of translation, including elongation and initiation.

Before discussing the details of the specific findings regarding these elementary steps, it is important to acknowledge certain limitations and considerations associated with the use of human PURE. Firstly, while it has been established that C9-RAN can proceed with minimal factors, it does not imply that such minimal factors are representative of physiological conditions. The results presented here do not rule out the possibility that the presence of additional factors might alter the properties of C9-RAN, either qualitatively or quantitatively. Indeed, it is worth noting true that translation efficiency in the human PURE was lower compared to that in the HeLa lysate system. Hence, it is plausible that the accumulation of translation products leading to toxicity may necessitate the involvement of supplementary factor(s).

Through the use of human PURE-IRES, which skips the complicated initiation reaction, we unequivocally demonstrated that the G_4_C_2_ repeat is translated by a defined set of eEFs. Furthermore, by enforcing translation initiation exclusively from the ATG codon immediately following the IRES, we were able to evaluate frameshifting from the GA (0) frame, as discussed in further detail below.

There have been debates regarding the requirement of a 5′ cap structure for C9-RAN^32,33,35,36^. Human PURE-cap experiments definitively established that the translation initiation mechanism for C9-RAN is indeed 5′ cap-dependent. These results not only corroborate previous studies that demonstrated 5′ cap-dependent C9-RAN using reporter assays, including those employing cell lysates^32,33^, but also suggest that even within a cellular environment where numerous factors are present, a specific set of eIFs could play a primary role in C9-RAN.

C9-RAN initiation, potentially involving canonical eIFs like eIF2, has been proposed^2,32,33,43^. Considering the specific nature of non-AUG translation in C9-RAN, it is plausible that eIF2A and eIF2D play roles in translation initiation. Indeed, previous studies implicated eIF2A and eIF2D as regulators of C9-RAN^35,43,44^, and another regulator of non-AUG translation, 5MP, has also been linked to RAN translation^59^. Although studies at the cellular level have examined the effects of translation factors on C9-RAN through knockdown or knockout experiments^35,43,44,59^, these methods may have had difficulty elucidating the direct effects of eIF2A, eIF2D, and other translational factors, likely due to the secondary effects associated with siRNA knockdown. It is noteworthy that the findings from human PURE-cap experiments indicate that eIF2A and eIF2D are not involved in the initiation of C9-RAN.

An intriguing and unresolved aspects of RAN translation, including C9-RAN, is the generation of translation products originating from multiple frames^2,60,61^. Previous studies conducted in cultured cells and cell lysates could not distinguish whether the translation of multiple frames results from ribosomal frameshifting or the presence of multiple translation initiation sites^33,39,42^. Through the dissection of C9-RAN using human PURE, we revealed that both mechanisms contribute to this phenomenon. Firstly, analysis utilizing human PURE-IRES uncovered the occurrence of frameshifting in the GA frame as in previous reports^33,39^(Fig. 3). Furthermore, this analysis demonstrated that longer G_4_C_2_ repeats enhance frameshifting in the GP frame. We propose that this enhancement, mediated by longer repeats, is attributed to the formation of G4RNA structures within the G_4_C_2_ repeats, as previous research indicates that G4RNA suppresses ribosomal translocation and promotes frameshifting^62^. It is worth noting that the frameshift efficiency in HeLa lysate was lower than that observed in human PURE-IRES. Therefore, the presence of specific factors in HeLa lysate may suppress ribosomal frameshifting. For instance, the abundance of RNA-binding proteins, such as TDP-43, known as RNA chaperones^63^, might lead to alterations in the G4RNA structure. Additionally, the interaction of the ribosome with Shiftless, a factor that suppresses -1 programmed frameshift^64^, may also influence the frameshift process.

Utilizing the human PURE-IRES system, we observed a ribosomal frameshift from the GA frame to the GP frame at a frequency of 3.5% (Fig. 3). In contrast, 5′ cap-dependent translation initiation using human PURE-cap exhibited higher efficiency in generating the GP frame, with a ratio of + 1 to 0 frame of approximately 17% (Fig. 4). This outcome suggests the involvement of an alternative mechanism that leads to the initiation at different sites, giving rise to the production of multiple frames, including the GP frame. Considering that human PURE-IRES and human PURE-cap differ solely in the presence of eIFs, approximately 80% (3.5%/17%) of the + 1 frame may undergo scanning-specific translation initiation mediated by the 5′ cap and canonical eIFs. Nevertheless, the initiation site for GP frame translation remains elusive. As the GP frame contains a stop codon preceding the G_4_C_2_ repeat (Fig. S1), it is plausible that initiation occurs from within the repeat sequence (Fig. 4E). Furthermore, G4RNA has been demonstrated to induce read-through of the stop codon^62^, implying that multiple translation mechanisms could contribute to GP frame translation.

Our analysis of C9-RAN translation using HeLa lysate revealed a translation efficiency comparable to canonical AUG codons for the GA frame (Fig. S4). This signifies a notably high efficiency of C9-RAN translation when compared to previous cell extract systems^32,33^. We attribute this to the remarkable translation promotion induced by the longer repeat RNAs, as demonstrated in Fig. 5. The variations in the extent of C9-RAN translation promotion by these repeat RNAs may be linked to the existence of specific translation-promoting factors within the cell extract system. A thorough investigation of these specific accelerators using the human PURE system will provide further insights into the defining characteristics of RAN translation.

Our analysis using human PURE-cap revealed a striking difference in repeat-length dependency. Cap-dependent C9-RAN was inhibited by longer repeat RNA in human PURE-cap. While previous reports have indicated that C9-RAN is enhanced in a repeat length-dependent manner in cell lysate translation systems and transfected cells^33,58^, our findings suggest the involvement of non-canonical translation factors in the repeat length-dependent stimulation of C9-RAN. In the human PURE-cap system, translation increase was observed for the GA frame up to (GGGGCC)_8_ repeats; however, translation efficiency was significantly reduced beyond 29 repeats, which are likely to form the G4RNA structure. Additionally, the mutation of the initiation codon CUG to the AUG codon in the GA frame also displayed inhibitory tendencies. Therefore, it is plausible that the inhibition of longer repeats-induced C9-RAN arises from elongation inhibition, potentially caused by the formation of G4RNA. Indeed, previous studies have demonstrated that non-AUG translation, including RAN translation, can be facilitated by downstream secondary structures^65,66^. Furthermore, the absence of RQC factors may also contribute to the repeat RNA-dependent translational repression observed in the human PURE system. Recent reports have highlighted that ribosomes experience stalling upon encountering repeat RNAs, leading to the activation of RQC^48^. This suggests that the presence of RQC factors may play a pivotal role in resolving the stalled ribosomes and promoting the efficient translation of repeat RNA.

If the characteristics of the repeat RNA itself contribute to the length-dependent inhibition, it is necessary to consider factors that bind to the RNA repeats. Alongside the RNA chaperone, which modulates RAN translation by interacting with repeat RNA and modifying its structure^63^, RNA helicases that unwind the higher-order structure of repeat RNA^67–69^ are also likely to be involved in the process. In HeLa lysate, RNA helicases such as DHX36 are responsible for unwinding G4RNA. DHX36 has been identified as a binding partner of G4RNA and is capable of unwinding its structure in an ATP-dependent manner, thereby rescuing ribosomal stalling on the G_4_C_2_ repeat^67,68^. However, knockdown or knockout of DHX36 only results in approximately 50% inhibition, even at G_4_C_2_ 70 repeats^67^, which falls significantly short of the inhibitory effect observed in human PURE-cap. Hence, it is reasonable to speculate that additional factors beyond DHX36 are involved in the elongation process of RAN translation. The dissection analysis conducted using human PURE, in conjunction with previous studies, suggests that translation of the G_4_C_2_ repeats represents a delicate balance between the promotion of noncanonical initiation and the inhibition of repeat elongation. Consequently, the presence of a novel regulator of RAN translation that enhances ribosomal translocation and exhibits ribosomal helicase activity may be implicated in C9-RAN within the cellular context.

Lastly, the human PURE system not only serves as an invaluable tool for studying C9-RAN, but also for investigating RAN translation involving other nucleotide repeats and, more broadly, for facilitating bottom-up approaches to noncanonical translation. Furthermore, the elucidation of the underlying mechanisms governing the fundamental process of RAN translation, as revealed by this reconstituted system, holds significant potential in the development of therapeutic strategies targeting neurodegenerative diseases associated with RAN translation.

## Methods

### Plasmids

To generate the HCV-IRES-(G_4_C_2_)_80_-Myc reporter plasmid, we employed the following procedure. The HCV-IRES region was amplified from the HCV-IRES sequence and introduced upstream of the previously published pcDNA^TM^5/FRT-T7-*C9orf72* intron1-(G_4_C_2_)_80_-Myc vector^70^ by means of HindIII and BssHII. In order to construct the *C9orf72* intron1-(G_4_C_2_)_80_-Nluc-3xFLAG, the Nluc-3xFLAG region was amplified from the Nluc sequence and inserted downstream of the aforementioned pcDNA^TM^5/FRT-T7-*C9orf72* intron1-(G_4_C_2_)_80_ vector using PstI. A similar strategy was employed to engineer the C9-RAN Nluc reporter with HCV-IRES. Repeat sequences of varying lengths were randomly obtained through PCR. The insertion of ATG, GGG, and near-cognate codons to the Nluc plasmid was achieved using standard cloning procedures and Gibson Assembly. Tables S3 and S4 provide a comprehensive list of the plasmids and oligonucleotides employed in this study.

### In vitro transcription

The reporter plasmids were linearized with XbaI (Takara). Subsequently, the linearized DNA was purified using the Wizard® SV Gel PCR purification kit (Promega). For the synthesis of 5′-capped mRNA, the reactions were carried out following previously established protocols^70^. Uncapped RNAs were transcribed in vitro using the CUGA T7 in vitro transcription kit (Nippon Gene). The size and quality of the resulting mRNAs were assessed through denaturing RNA gel electrophoresis.

### Human PURE in vitro translation

The HCV-IRES-dependent in vitro translation was conducted according to previously described methods^51^. In brief, for the luminescence assay of nano luciferase, a mixture of human PURE cocktail (4.5 µL) and reporter DNAs (0.5 µL, 15 nM) was incubated at 32 °C for 3 h, followed by termination through incubation on ice. The subsequent steps were performed using 96-well plates. A volume of 2 µL of the samples was diluted in Glo lysis buffer (Promega) and incubated in a 1:1 ratio for 3 min in the dark with NanoGlo substrate, freshly diluted a 1:50 ratio in NanoGlo buffer (Promega), with shaking. Luminescence was measured using a Varioskan LUX Multimode Microplate Reader (Thermo Fisher Scientific). For western blotting, the same procedure was followed, except that 6 µL of samples were used. The 6 µL reactions were mixed with 15 µL of 4xSDS sample buffer (240 mM Tris–HCl pH 6.8, 40% (v/v) glycerol, 0.01% (w/v) bromophenol blue, 7% (w/v) SDS, 10% (v/v) of 2-mercaptoethanol) and heated at 70 °C for 10 min. Subsequently, 20 µL of the samples were loaded onto a 13% SDS–polyacrylamide gel for electrophoresis and subsequent western blotting analysis.

The cap-dependent in vitro translation was conducted following the established protocol^52^. Briefly, the human PURE-cap cocktail (3.6 µL) was combined with 0.4 µL of reporter mRNAs (final concentration, 60 nM) and incubated at 32 °C for 6 h. The samples were analyzed using the same approach as the HCV-IRES-dependent human PURE translation.

In experiments involving the addition of eIF2A and eIF2D, the human PURE cocktail was prepared to replace eIF2 at a final concentration of 0.5 µM (eIF2(−) condition). The same procedure was also conducted under eIF2(+) conditions.

### HeLa lysate in vitro translation

The in vitro translation system derived from HeLa S3 cells was as previously described^71^. The HCV-IRES-dependent translation was assessed using the same procedure as the human PURE translation after incubation at 32 °C for 2 h. In the cap-dependent in vitro translation, reporter mRNAs were added at a concentration of 3 nM and incubated at 32 °C for 1 h. The samples were analyzed following the same method as the human PURE in vitro translation.

### Western blotting

The samples were separated on 13% SDS–polyacrylamide gels and transferred onto PVDF membranes. The membranes were blocked with 2% (w/v) skim milk in TBS-T. Subsequently, the membranes were incubated with primary antibodies listed in Table S5. After washes with TBS-T, the membranes were incubated with HRP-conjugated anti-mouse IgG (Sigma-Aldrich). Chemiluminescence signals were detected using an LAS4000 (FujiFilm).

### Recombinant protein expression and purification

Recombinant GST-eIF2D and GST-eIF2A-His were produced in Rosetta 2(DE3) *E. coli* (Sigma # 71397-4) using 2xYT media supplemented with 20 μg/ml chloramphenicol and 100 μg/ml carbenicilin. Isopropyl-β–D-thiogalactopyranoside (final concentration, 1 mM) was added to induce the expression of the eIF2D and eIF2A proteins at an optical density at 600 nm of 0.5–0.8. These cells were cultured at 16 °C for 18–20 h. After washing with a PBS buffer (nacalai), the bacterial pellet was kept − 80 °C. Recombinant eIF2D was purified using N-terminal GST-tag. The frozen cell pellets were resuspended in a buffer (20 mM HEPES–KOH pH 7.5, 500 mM KCl, 2 mM DTT, 0.1%(v/v) Triton-X-100, 10%(v/v) Glycerol and cOmplete EDTA-free protease inhibitor cocktail tablet (Roche)) and lysed by sonication. Cell lysates were cleared by centrifugation for 45 min at 20,000 × *g*. The supernatant was incubated with Glutathione Sepharose 4B (Cytiva) for 1 h at 4 °C. The resin was washed with the same buffer and a high-salt wash buffer (20 mM HEPES–KOH pH 7.5, 1 M KCl, 2 mM DTT, 0.1%(v/v) Triton-X-100, 10%(v/v) Glycerol) Then, the wash buffer was exchanged for Precission cleavage buffer (50 mM Tris–HCl pH 7.5, 500 mM NaCl, 10%(v/v) Glycerol, 0.1%(v/v) Triton-X-100, 1 mM EDTA, 1 mM DTT). GST-tag was cleaved on-column by Precission protease (Cytiva) for 16 h at 4 °C. The flow-through fraction was applied onto a PD-10 column (GE Healthcare) equilibrated with a stored buffer (20 mM HEPES–KOH pH 7.5, 100 mM KCl, 10%(v/v) Glycerol). After elution with 3.5 mL of the same buffer, the eluate was concentrated and stored at − 80 °C. In addition to the GST-based purification, recombinant eIF2A was further purified by using C-terminal His-tag. After GST-tag cleavage, flow-through fraction including eIF2A was applied onto a Ni–NTA resin (Qiagen), and the resin was washed with another buffer (20 mM HEPES–KOH, 100 mM KCl, 10%(v/v) Glycerol, 1 mM DTT, 20 mM imidazole, 1 mM ATP, 1 mM MgCl_2_). eIF2A was eluted with elution buffer (20 mM HEPES–KOH pH 7.5, 100 mM KCl, 10%(v/v) Glycerol, 300 mM imidazole). Finally, eIF2A was desalted and buffer exchanged into the eIF2D stored buffer, and concentrated.

### Quantification and statistical analysis

All statistical analyses were performed using custom R code. The presented quantitative data represent the mean ± S.D. from a minimum of three independent experiments.

### Supplementary Information

Supplementary Information 1.

Supplementary Information 2.

Supplementary Information 3.

Supplementary Information 4.

Supplementary Information 5.

Supplementary Information 6.

## Data Availability

Data in this manuscript have been uploaded to the Mendeley Dataset public repository (https://doi.org/10.17632/ycnm3f8gsf.1).

## References

[1] Hannan AJ (2018). Tandem repeats mediating genetic plasticity in health and disease. Nat. Rev. Genet..

[2] Malik I, Kelley CP, Wang ET, Todd PK (2021). Molecular mechanisms underlying nucleotide repeat expansion disorders. Nat. Rev. Mol. Cell Biol..

[3] Zu T (2011). Non-ATG-initiated translation directed by microsatellite expansions. Proc. Natl. Acad. Sci. U. S. A..

[4] Ash PEA (2013). Unconventional translation of C9ORF72 GGGGCC expansion generates insoluble polypeptides specific to c9FTD/ALS. Neuron.

[5] Zu T (2017). RAN translation regulated by muscleblind proteins in myotonic dystrophy type 2. Neuron.

[6] Mori K (2013). The C9orf72 GGGGCC repeat is translated into aggregating dipeptide-repeat proteins in FTLD/ALS. Science.

[7] Bañez-Coronel M (2015). RAN translation in huntington disease. Neuron.

[8] Soragni E (2018). Repeat-associated non-ATG (RAN) translation in Fuchs’ endothelial corneal dystrophy. Invest. Ophthalmol. Vis. Sci..

[9] Todd PK (2013). CGG repeat-associated translation mediates neurodegeneration in fragile X tremor ataxia syndrome. Neuron.

[10] DeJesus-Hernandez M (2011). Expanded GGGGCC hexanucleotide repeat in noncoding region of C9ORF72 causes chromosome 9p-linked FTD and ALS. Neuron.

[11] Renton AE (2011). A hexanucleotide repeat expansion in C9ORF72 is the cause of chromosome 9p21-linked ALS-FTD. Neuron.

[12] Gendron TF (2013). Antisense transcripts of the expanded C9ORF72 hexanucleotide repeat form nuclear RNA foci and undergo repeat-associated non-ATG translation in c9FTD/ALS. Acta Neuropathol..

[13] Zu T (2013). RAN proteins and RNA foci from antisense transcripts in C9ORF72 ALS and frontotemporal dementia. Proc. Natl. Acad. Sci. U. S. A..

[14] Jovičič A (2015). Modifiers of C9orf72 dipeptide repeat toxicity connect nucleocytoplasmic transport defects to FTD/ALS. Nat. Neurosci..

[15] Zhang YJ (2018). Poly(GR) impairs protein translation and stress granule dynamics in C9orf72-associated frontotemporal dementia and amyotrophic lateral sclerosis. Nat. Med..

[16] Zhang YJ (2016). C9ORF72 poly(GA) aggregates sequester and impair HR23 and nucleocytoplasmic transport proteins. Nat. Neurosci..

[17] Mizielinska S (2014). C9orf72 repeat expansions cause neurodegeneration in Drosophila through arginine-rich proteins. Science.

[18] Khosravi B (2017). Cytoplasmic poly-GA aggregates impair nuclear import of TDP-43 in C9orf72 ALS/FTLD. Hum. Mol. Genet..

[19] May S (2014). C9orf72 FTLD/ALS-associated Gly-Ala dipeptide repeat proteins cause neuronal toxicity and Unc119 sequestration. Acta Neuropathol..

[20] Mori K (2013). Bidirectional transcripts of the expanded C9orf72 hexanucleotide repeat are translated into aggregating dipeptide repeat proteins. Acta Neuropathol..

[21] Schuller AP, Green R (2018). Roadblocks and resolutions in eukaryotic translation. Nat. Rev. Mol. Cell Biol..

[22] Sonenberg N, Hinnebusch AG (2009). Regulation of translation initiation in eukaryotes: Mechanisms and biological targets. Cell.

[23] Jackson RJ, Hellen CUT, Pestova TV (2010). The mechanism of eukaryotic translation initiation and principles of its regulation. Nat. Rev. Mol. Cell Biol..

[24] Ingolia NT, Lareau LF, Weissman JS (2011). Ribosome profiling of mouse embryonic stem cells reveals the complexity and dynamics of mammalian proteomes. Cell.

[25] Ingolia NT, Ghaemmaghami S, Newman JRS, Weissman JS (2009). Genome-wide analysis in vivo of translation with nucleotide resolution using ribosome profiling. Science.

[26] Chen J (2020). Pervasive functional translation of noncanonical human open reading frames. Science.

[27] Kearse MG, Wilusz JE (2017). Non-AUG translation: A new start for protein synthesis in eukaryotes. Genes Dev..

[28] Dmitriev SE (2010). GTP-independent tRNA delivery to the ribosomal P-site by a novel eukaryotic translation factor. J. Biol. Chem..

[29] Starck SR (2012). Leucine-tRNA initiates at CUG start codons for protein synthesis and presentation by MHC class I. Science.

[30] Skabkin MA (2010). Activities of Ligatin and MCT-1/DENR in eukaryotic translation initiation and ribosomal recycling. Genes Dev..

[31] Yang Y, Wang Z (2019). IRES-mediated cap-independent translation, a path leading to hidden proteome. J. Mol. Cell Biol..

[32] Green KM (2017). RAN translation at C9orf72-associated repeat expansions is selectively enhanced by the integrated stress response. Nat. Commun..

[33] Tabet R (2018). CUG initiation and frameshifting enable production of dipeptide repeat proteins from ALS/FTD C9ORF72 transcripts. Nat. Commun..

[34] Boivin M (2020). Reduced autophagy upon C9ORF72 loss synergizes with dipeptide repeat protein toxicity in G4C2 repeat expansion disorders. EMBO J..

[35] Sonobe Y (2018). Translation of dipeptide repeat proteins from the C9ORF72 expanded repeat is associated with cellular stress Neurobiol. Dis..

[36] Cheng W (2018). C9ORF72 GGGGCC repeat-associated non-AUG translation is upregulated by stress through eIF2α phosphorylation. Nat. Commun..

[37] Lampasona A, Almeida S, Gao FB (2021). Translation of the poly(GR) frame in C9ORF72-ALS/FTD is regulated by cis-elements involved in alternative splicing. Neurobiol. Aging.

[38] van’t Spijker HM (2021). Ribosome profiling reveals novel regulation of C9ORF72 GGGGCC repeat-containing RNA translation. RNA.

[39] McEachin ZT (2020). Chimeric peptide species contribute to divergent dipeptide repeat pathology in c9ALS/FTD and SCA36. Neuron.

[40] Gaspar C (2000). CAG tract of MJD-1 may be prone to frameshifts causing polyalanine accumulation. Hum. Mol. Genet..

[41] Girstmair H (2013). Depletion of cognate charged transfer RNA causes translational frameshifting within the expanded CAG stretch in huntingtin. Cell Rep..

[42] Wright SE (2022). CGG repeats trigger translational frameshifts that generate aggregation-prone chimeric proteins. Nucleic Acids Res..

[43] Green KM, Miller SL, Malik I, Todd PK (2022). Non-canonical initiation factors modulate repeat-associated non-AUG translation. Hum. Mol. Genet..

[44] Sonobe Y (2021). A *C. elegans* model of C9orf72-associated ALS/FTD uncovers a conserved role for eIF2D in RAN translation. Nat. Commun..

[45] Radwan M (2020). Arginine in C9ORF72 dipolypeptides mediates promiscuous proteome binding and multiple modes of toxicity. Mol. Cell Proteomics.

[46] Park J (2021). ZNF598 co-translationally titrates poly(GR) protein implicated in the pathogenesis of C9ORF72-associated ALS/FTD. Nucleic Acids Res..

[47] Kriachkov V (2023). Arginine-rich C9ORF72 ALS proteins stall ribosomes in a manner distinct from a canonical ribosome-associated quality control substrate. J. Biol. Chem..

[48] Tseng Y-J (2023). Ribosomal quality control factors inhibit repeat-associated non-AUG translation from GC-rich repeats. bioRxiv.

[49] Sauert M, Temmel H, Moll I (2015). Heterogeneity of the translational machinery: Variations on a common theme. Biochimie.

[50] Shimizu Y (2001). Cell-free translation reconstituted with purified components. Nat. Biotechnol..

[51] Machida K (2014). A translation system reconstituted with human factors proves that processing of encephalomyocarditis virus proteins 2A and 2B occurs in the elongation phase of translation without eukaryotic release factors. J. Biol. Chem..

[52] Machida K (2018). Dynamic interaction of poly(A)-binding protein with the ribosome. Sci. Rep..

[53] Abe T, Nagai R, Imataka H, Takeuchi-Tomita N (2020). Reconstitution of yeast translation elongation and termination in vitro utilizing CrPV IRES-containing mRNA. J. Biochem..

[54] Lancaster AM, Jan E, Sarnow P (2006). Initiation factor-independent translation mediated by the hepatitis C virus internal ribosome entry site. RNA.

[55] Wang S (2021). Nuclear export and translation of circular repeat-containing intronic RNA in C9ORF72-ALS/FTD. Nat. Commun..

[56] Almeida S (2019). Production of poly(GA) in C9ORF72 patient motor neurons derived from induced pluripotent stem cells. Acta Neuropathol..

[57] Sellier C (2017). Translation of expanded CGG repeats into fmrpolyg is pathogenic and may contribute to fragile X tremor ataxia syndrome. Neuron.

[58] Kearse MG (2016). CGG repeat-associated non-AUG translation utilizes a cap-dependent scanning mechanism of initiation to produce toxic proteins. Mol. Cell.

[59] Singh CR (2021). Human oncoprotein 5MP suppresses general and repeat-associated non-AUG translation via eIF3 by a common mechanism ll Human oncoprotein 5MP suppresses general and repeat-associated non-AUG translation via eIF3 by a common mechanism. Cell Rep..

[60] Cleary JD, Ranum LP (2017). New developments in RAN translation: Insights from multiple diseases. Curr. Opin. Genet. Dev..

[61] Cleary JD, Pattamatta A, Ranum LPW (2018). Repeat-associated non-ATG (RAN) translation. J. Biol. Chem..

[62] Yu C-H, Teulade-Fichou M-P, Olsthoorn RCL (2014). Stimulation of ribosomal frameshifting by RNA G-quadruplex structures. Nucleic Acids Res..

[63] Ishiguro T (2017). Regulatory role of RNA chaperone TDP-43 for RNA misfolding and repeat-associated translation in SCA31. Neuron.

[64] Wang X (2019). Regulation of HIV-1 Gag-Pol expression by shiftless, an inhibitor of programmed -1 ribosomal frameshifting. Cell.

[65] Kozak M (1990). Downstream secondary structure facilitates recognition of initiator codons by eukaryotic ribosomes. Proc. Natl. Acad. Sci. U. S. A..

[66] Kearse MG (2019). Ribosome queuing enables non-AUG translation to be resistant to multiple protein synthesis inhibitors. Genes Dev..

[67] Tseng Y-J (2021). The RNA helicase DHX36/G4R1 modulates C9orf72 GGGGCC hexanucleotide repeat- associated translation. J. Biol. Chem..

[68] Liu H (2021). A helicase unwinds hexanucleotide repeat RNA G-quadruplexes and facilitates repeat-associated non-AUG translation. J. Am. Chem. Soc.

[69] Cheng W (2019). CRISPR-Cas9 screens identify the RNA helicase DDX3X as a repressor of C9ORF72 (GGGGCC)n repeat-associated non-AUG translation. Neuron.

[70] Fujino Y (2023). FUS regulates RAN translation through modulating the G-quadruplex structure of GGGGCC repeat RNA in C9orf72-linked ALS/FTD. Elife.

[71] Mikami S (2008). A human cell-derived in vitro coupled transcription/translation system optimized for production of recombinant proteins. Protein Expr. Purif..

