# Supplementary material for: Reconstitution of C9orf72 GGGGCC repeat-associated non-AUG translation with purified human translation factors
Source: Sci Rep. 2023 Dec 20;13:22826. doi: 10.1038/s41598-023-50188-z (PMC10739749; doi:10.1038/s41598-023-50188-z)

Supporting Information

Reconstitution of *C9orf72* GGGGCC repeat-associated non-AUG translation with purified human translation factors

Hayato Ito, Kodai Machida, Mayuka Hasumi, Morio Ueyama, Yoshitaka Nagai, Hiroaki Imataka and Hideki Taguchi

*Correspondence author: Hideki Taguchi
Email: taguchi@bio.titech.ac.jp

This PDF file includes:

Supplementary Figures S1 to S6
Supplementary Tables S1 to S5
Unprocessed images for each Figure

5′……agtactcgc**tga**ggg**tga**acaagaaaagacc**tgataa**agatt
aaccagaagaaaacaaggagggaaacaaccgcagcctg**tag**caagct
ctggaactcaggagtcgcgcgc**t** **aG**G GGCCGGGGCCGGGGCC……3′

Fig. S1: The sequence of the first intron of *C9orf72*.

The GGGGCC repeat is represented in a shaded gray box. The CTG codon, positioned at -24 nucleotides (magenta box) from the repeat sequence, was previously identified as the start codon for the GA frame (32-35). The AGG codon, overlapping with the repeat sequence (green box), was previously identified as the start codon for the GR frame (34). Stop codons are present in all frames (red (GP frame), brown (GR frame), purple (GA frame)), sequentially arranged from the 5' to 3' direction.

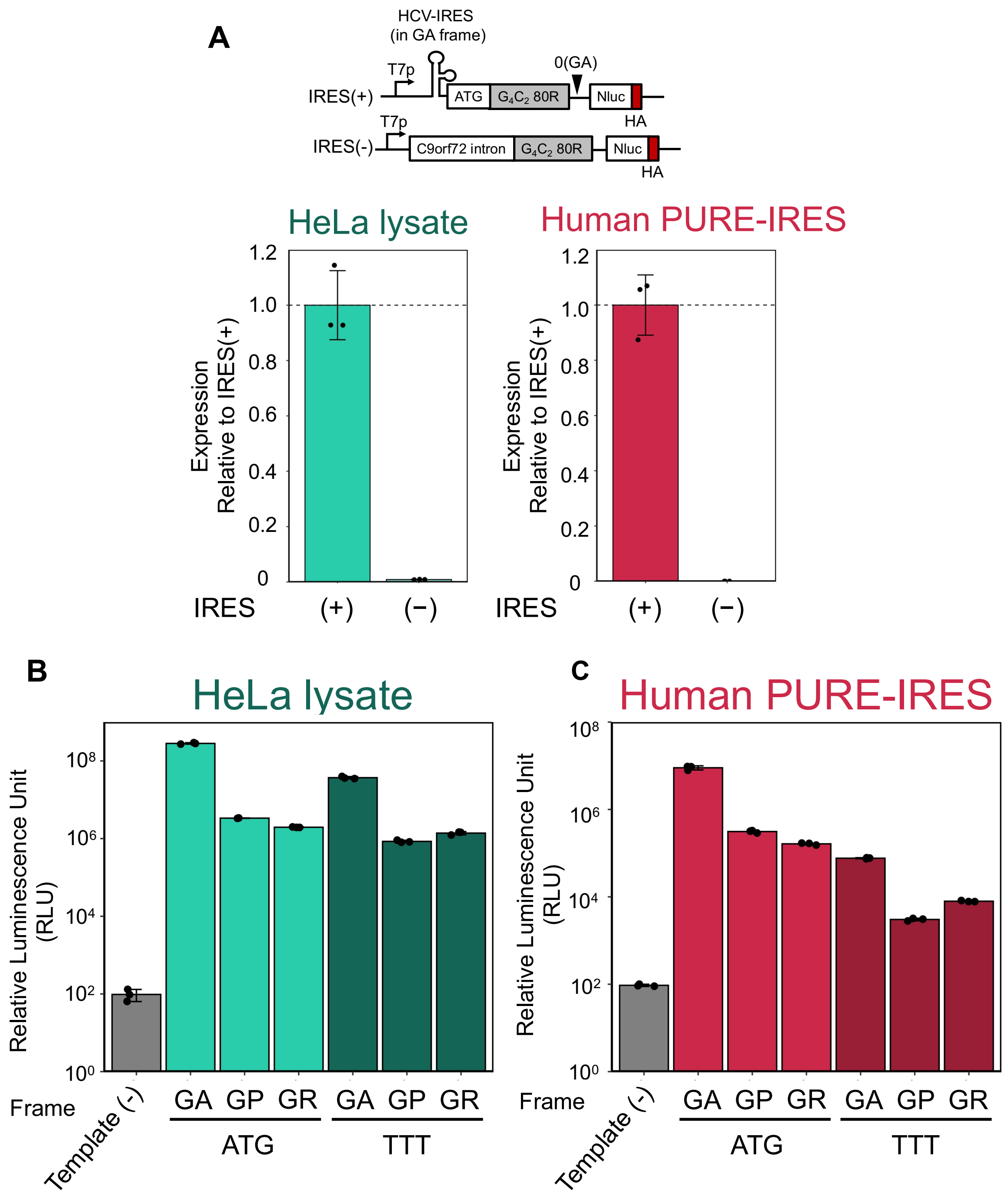

Fig. S2: HCV IRES-mediated translation using either HeLa lysate or human PURE-IRES.

(*A*) The expression of IRES(-)-$(G_4C_2)_{80}$-Nluc and IRES(+) reporters in each in vitro translation system.

(*B*, *C*) The absolute values of relative luminescence units (RLU) in HeLa lysate (*B*) or human PURE-IRES (*C*).

Error bars indicate standard deviations (±SD) derived from three technical replicates.

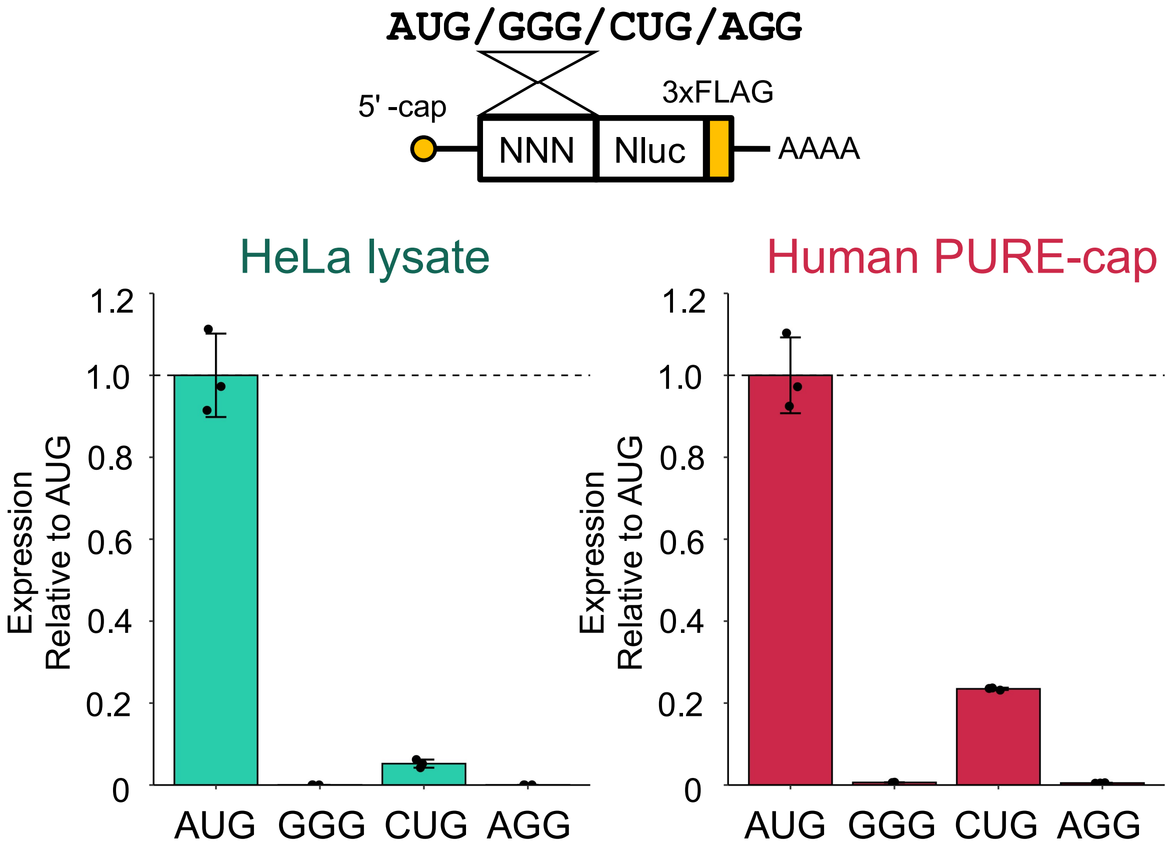

Fig. S3: Characteristics of non-AUG translation initiation in either HeLa lysate or human PURE-cap.

Relative expression levels of non-AUG translation reporters in each in vitro translation system are shown. The values were normalized with respect to that of AUG-Nluc. Error bars indicate ±SD from three technical replicates.

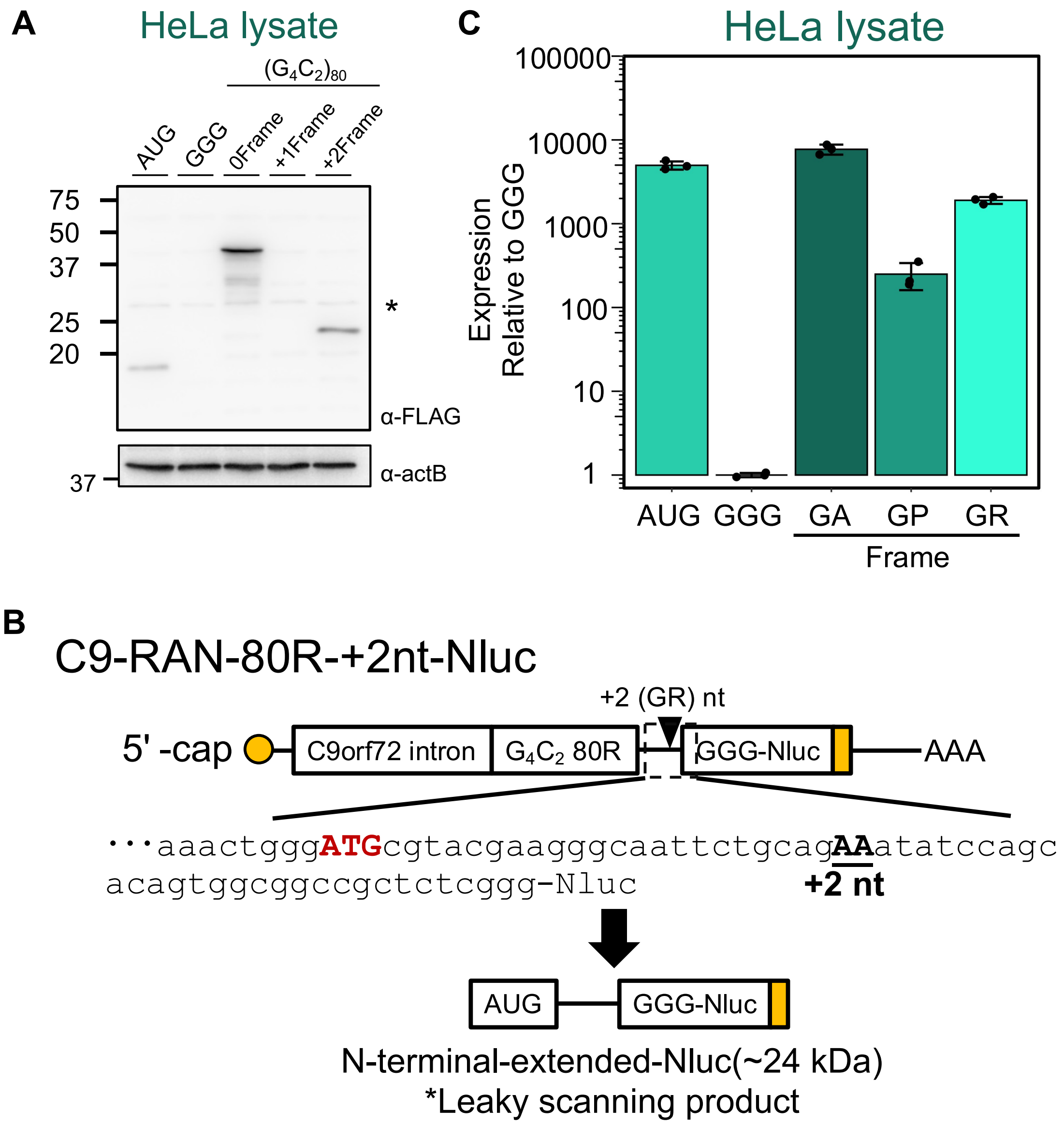

Fig. S4: Cap-mediated translation of the C9-RAN reporters in the HeLa lysate.

(*A*) An anti-FLAG western blot of the C9-RAN reporter plasmids expressed in the HeLa lysate is shown. An asterisk (*) denotes a non-specific band.

(*B*) The nucleotide sequence downstream of the G_4C_2 repeat, highlighting an ATG codon potentially associated with a leaky scanning product in the C9-RAN-80R-+2 nt-Nluc reporter.

(*C*) Relative expression levels of C9-RAN reporters are presented. The values were normalized to that of GGG-Nluc. Error bars indicate ±SD from three technical replicates.

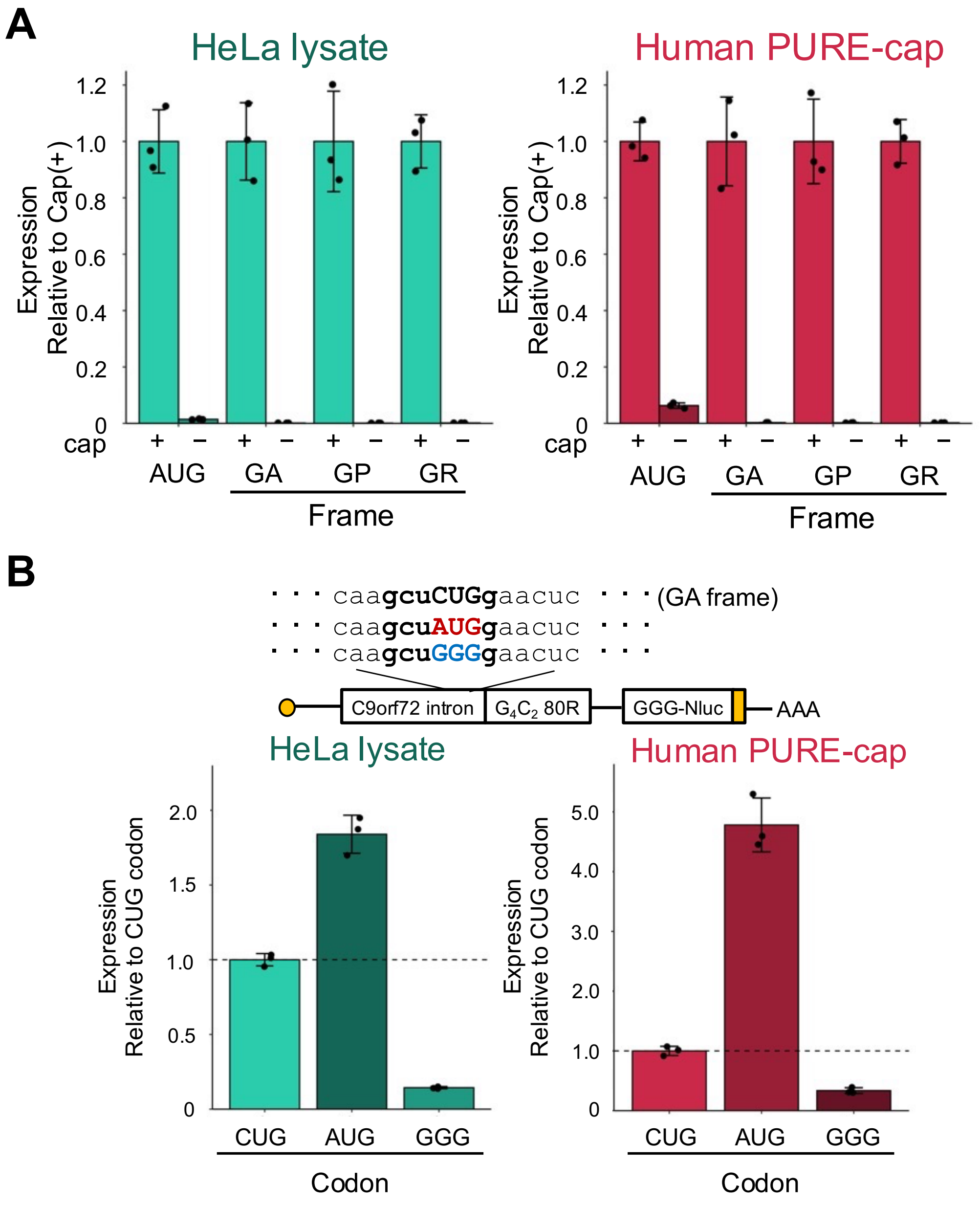

Fig. S5: C9-RAN initiates translation from the upstream of the repeat sequence in a 5'-cap-dependent manner.

(*A*) 5'-cap-dependecy: The values were normalized to those of cap(+) C9-RAN reporters.

(*B*) CUG in C9 intron dependency: The values were normalized to those of the CUG codon in the GA frame located upstream of the G_4C_2 repeat.

Error bars indicate ±SD from three technical replicates.

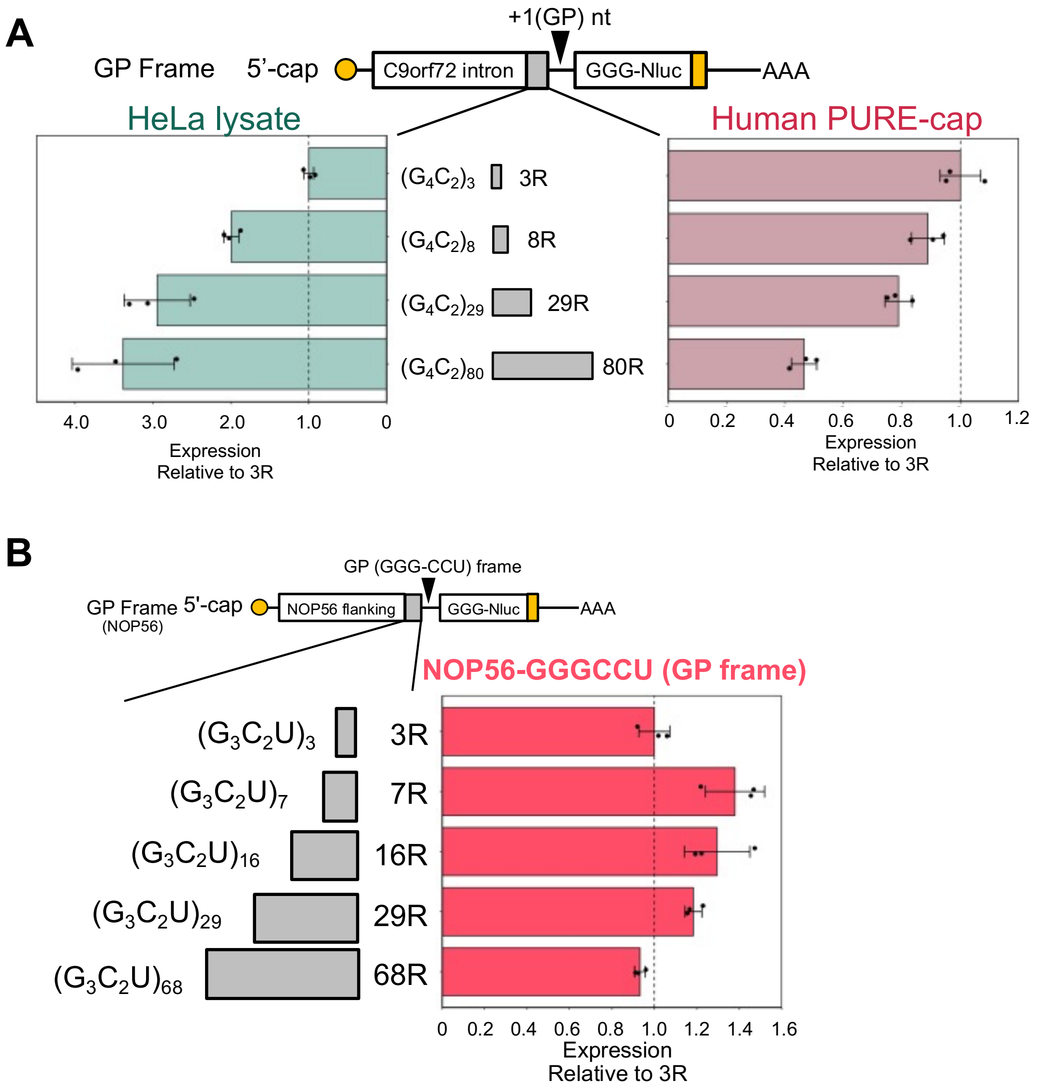

Fig. S6: Repeat-length dependent inhibition of GP frame in the human PURE-cap system.

(*A*) Relative expression levels of C9-RAN translation reporters with varying numbers of repeats in each in vitro translation system in the GP frame are presented. The values were normalized to those of 3R-Nluc. Error bars indicate ±SD from three technical replicates.

(*B*) Relative expression levels of NOP56-RAN translation reporters with varying numbers of repeats in the human PURE system in the GP frame. The values were normalized to those of 3R-Nluc. Error bars indicate ±SD from three technical replicates.

Unprocessed image

Fig. 2C
Western Blot(α-Myc)

Fig. 3B
Western Blot (α-actB)

Western Blot (α-HA)

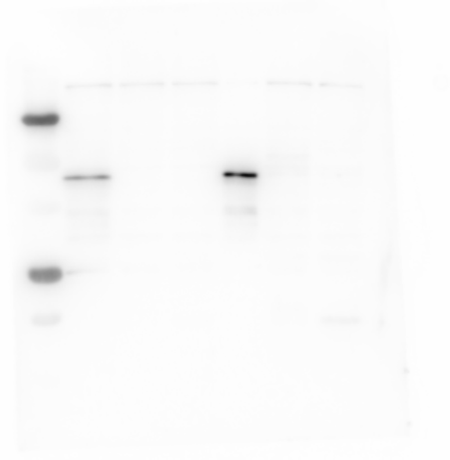

Fig. 3D
Western Blot (α-HA)

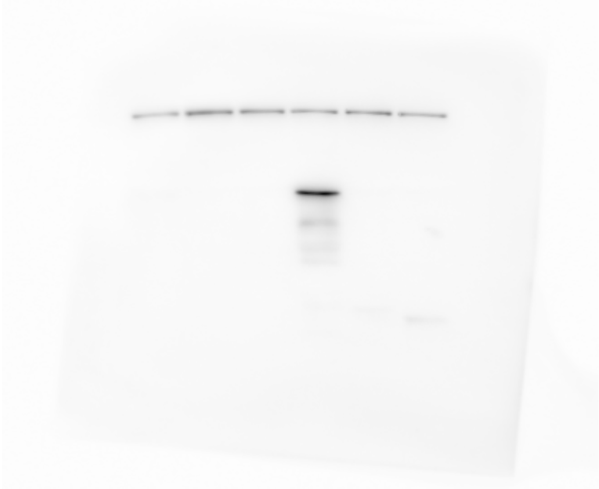

Fig. 4C
Western Blot (α-FLAG)

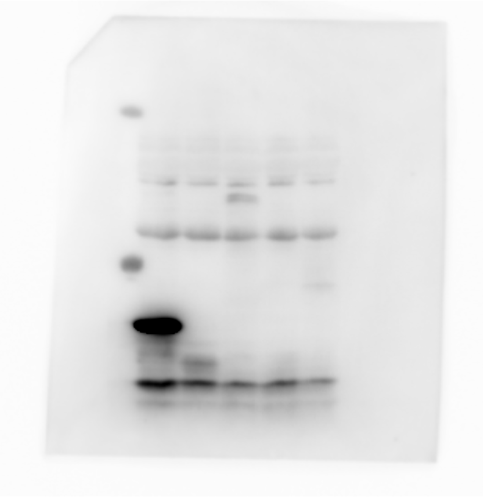

Fig. S4A
Western Blot (α-actB)
*Black line: Blot edge, Dashed line: Cropped region

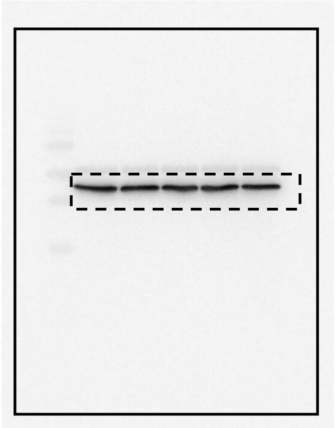

Fig. S4A
Western Blot (α-FLAG)
*Black line: Blot edge, Dashed line: Cropped region

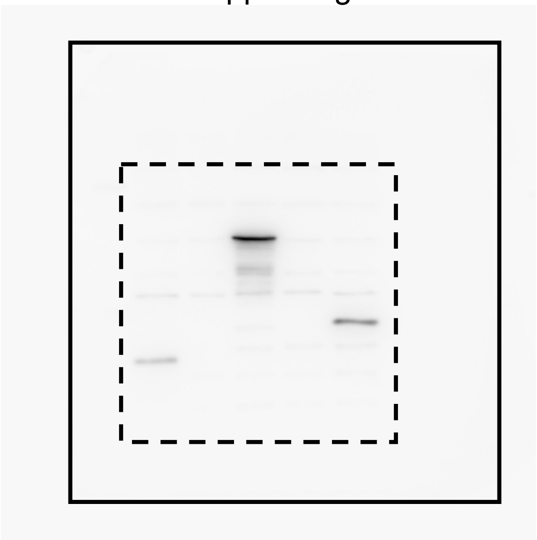

Supplement: Supplementary file 1 — Supplementary Information 1. [file 41598_2023_50188_MOESM1_ESM.pdf]
